# Effects of Polysaccharides from Different Species of *Dendrobium* (*Shihu*) on Macrophage Function

**DOI:** 10.3390/molecules18055779

**Published:** 2013-05-17

**Authors:** Lan-Zhen Meng, Guang-Ping Lv, De-Jun Hu, Kit-Leong Cheong, Jing Xie, Jing Zhao, Shao-Ping Li

**Affiliations:** State Key Laboratory of Quality Research in Chinese Medicine, and Institute of Chinese Medical Sciences, University of Macau, Macao SAR, China

**Keywords:** *Dendrobium*, polysaccharides, RAW 264.7 macrophages, immune function

## Abstract

*Dendrobium* spp. are precious medicinal plants, used in China for thousands of years as health foods and nutrients. Polysaccharides are the main effective ingredients in *Dendrobium* plants. In this study, the chemical characteristics and the effects of crude polysaccharides (CPs) from five species of *Dendrobium* on macrophage function were investigated and compared *in vitro* for the first time. Chemical characteristic studies showed that CPs from different species of *Dendrobium* were diverse, displaying widely varied Mw distributions and molar ratios of monosaccharides. Their effects on macrophage functions, such as promoting phagocytosis, release of NO and cytokines IL-1α, IL-6, IL-10 and TNF-α, were also different. Moreover, CPs from *D.* *officinale*, especially collected from Yunnan Province, exerted the strongest immunomodulatory activities and could be explored as a novel potential functional food. The diverse chemical characteristics of CPs from different species of *Dendrobium* might contribute to their varied effects on macrophage functions, which should be further investigated.

## 1. Introduction

The genus *Dendrobium* is one of the largest groups of the family *Orchidaceae*. There are 78 species of *Dendrobium* plants found in China and about 30 of them, well known as *Shihu*, are eaten or used as or folk medicines for antipyretic, eye-benefitting and immunoregulatory purposes [1]. In China, more than fifty *Dendrodium*-based health food products have been approved by the State Food and Drug Administration. Besides phenols, alkaloids, coumarins, terpenes and flavonoids [1,2,3], polysaccharides are also considered as one of the main active ingredients in *Dendrobium* plants [4]. Polysaccharides obtained from different species of *Dendrobium*, such as *D. huoshanense* [5,6], *D. denneanum* [7,8,9,10], *D. nobile* [11,12,13,14,15,16] and *D. candidum* [17], have been shown obvious antioxidant, immunostimulating, anti-tumor and anti-mutagenic activities. Interestingly, previous reports indicated that polysaccharides from different *Dendrobium* species obviously differed in their compositional monosaccharides [18], carbohydrase enzymatic digestion properties and profiles [19,20]. These diverse chemical characteristics of the polysaccharides from *Dendrobium* may be correlated with their health benefits.

**Figure 1:**
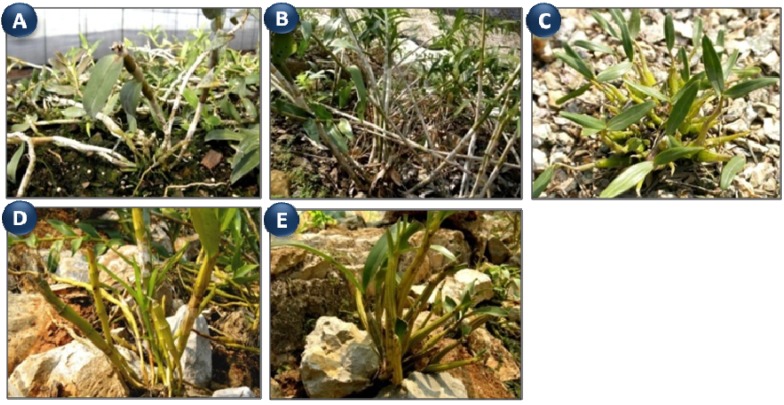
The plants of (**A**) *Dendrobium officinale*, (**B**) *D. fimbriatum*, (**C**) *D. huoshanense*, (**D**) *D. nobile* and (**E**) *D. chrysotoxum*.

In this study, the effects of crude polysaccharides (CPs) from five species of *Dendrobium*, including *D. officinale* (DO), *D. fimbriatum*(DF), *D. huoshanense* (DH), *D. nobile*(DN) and *D. chrysotoxum* (DC) (Figure 1), on macrophages were investigated and compared for the first time. The bioactivity results combined with their chemical study should be helpful to elucidate their medical value and for quality control of the polysaccharides from *Dendrobium*.

## 2. Results

### 2.1. Characteristics of Polysaccharides from Dendrobium

The yields of CPs of the five species of *Dendrobium* varied greatly, from 3.6%–13.4% (w/w). In brief, DC from Yunnan and DO from Zhejiang showed the lowest and highest yields, respectively. The total carbohydrate contents in CPs from different *Dendrobium* were 70.8%–93.0% (w/w, Table 1).

**Table 1:** Yield, carbohydrate content, molecular weight and compositional monosaccharide of crude polysaccharides from *Dendrobium.*

Polysaccharides	Yield (%, g/g)	Total Carbohydrates (%, g/g)	Molecular Weight		Compositional Monosaccharides	
			Peak 1	Peak 2	Monosaccharide	Molar ratio
DOYN	9.6	70.8	1.5 × 10^6^	7.1 × 10^4^	Man: Glu: Gal	100:206:5
DOAH	5.8	84.2	1.2 × 10^5^		Man: Glu: Gal	100:44:02
DOZJ	13.4	82.2	4.9 × 10^4^		Man: Glu: Gal	100:38:01
DFYN	6	78	3.2 × 10^6^	9.2 × 10^4^	Man: Glu: Gal	100:523:2
DHAH	7.4	93	2.2 × 10^5^		Man: Glu: Gal	100:53:02
DNYN	9.6	90	5.3 × 10^5^		Man: Glu: Gal	100:133:2
DCYN	3.6	92.4	3.3 × 10^6^	1.1 × 10^5^	Man: Glu	100:614

^a^ DOYN, DOAH, DOZJ, *D. officinale* from Yunnan, Anhui and Zhejiang, respectively; DFYN, *D.* *fimbriatum* from Yunnan; DHAH, *D.* *huoshanense* from Anhui, DNYN, *D.* *nobile* from Yunnan; DCYN, *D.* *chrysotoxum* from Yunnan. ^b^ Man = mannose; Glu = glucose; Gal = galactose.

**Figure 2:**
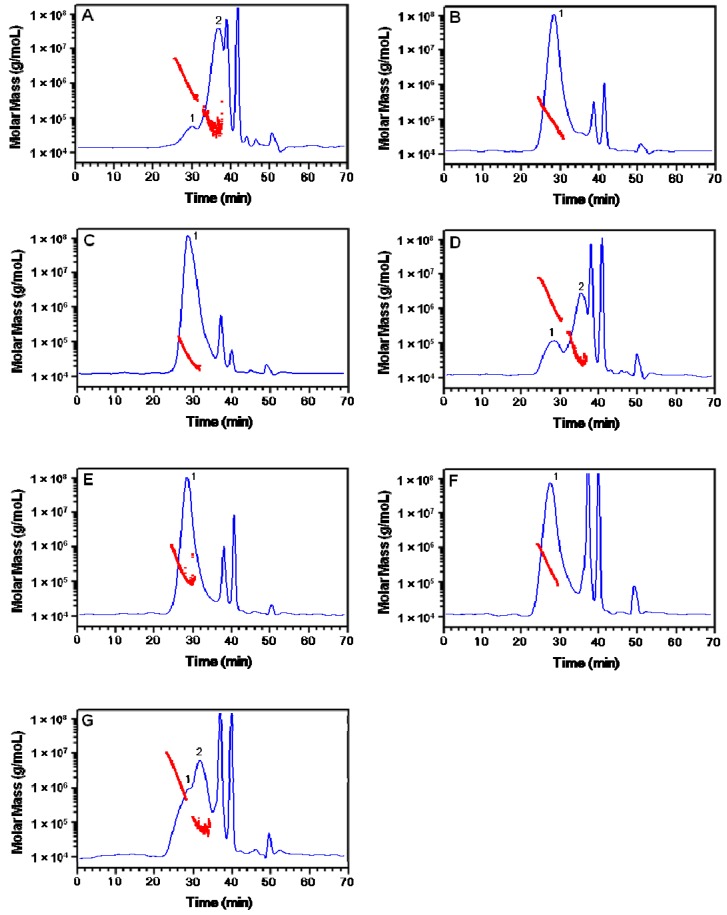
HPSEC-RID profiles with molecular weight distribution of polysaccharides from *Dendrobium officinale* from (**A**) Yunnan, (**B**) Anhui, (**C**) Zhejiang (**D**) *D. fimbriatum* from Yunnan, (**E**) *D. huoshanense* from Anhui, (**F**) *D. nobile* and (**G**) *D. chrysotoxum* from Yunnan.

Their profiles and average molecular weights (Mw), as well as Mw distribution, were determined using HPSEC-MALLS-RI detection. Figure 2 shows that all polysaccharides from *Dendrobium* usually had 1–2 fractions with more than 5 × 10^4^ Mw (Table 1). The other peaks might be from the buffer salt or saccharides degraded from CPs, which were too small to be detected well by the MALLS detector (data not shown).

**Figure 3:**
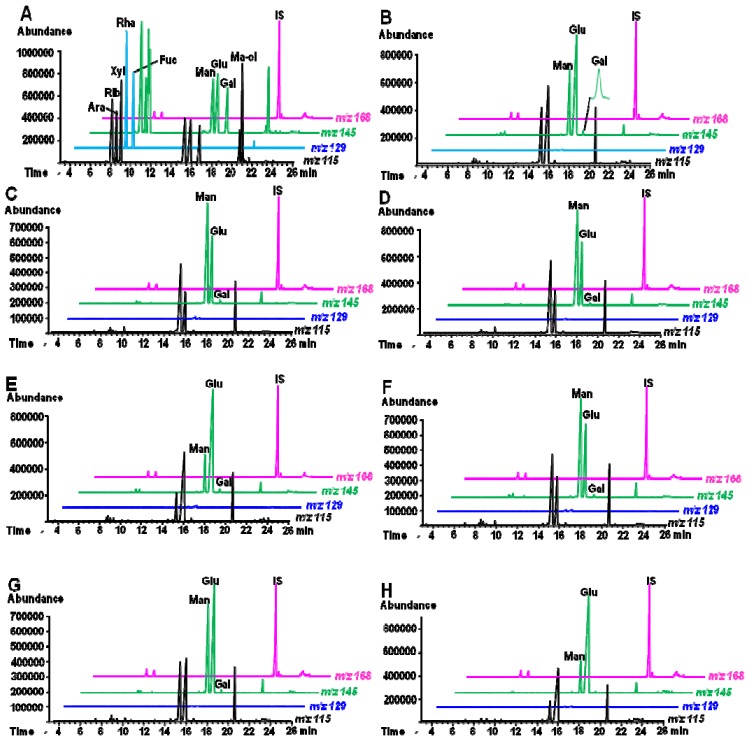
Typical SIM chromatograms of (**A**) mixed standards, and polysaccharides of *Dendrobium officinale* from (**B**) Yunnan, (**C**) Anhui, (**D**) Zhejiang, (**E**) *D. fimbriatum* from Yunnan, (**F**) *D. huoshanense* from Anhui, (**G**) *D. nobile* and (**H**) *D. chrysotoxum* from Yunnan.

^a^ Ara, arabinose; Rib, ribose; Xyl, xylose; Rha, rhamnose; Fuc, Fucose; Man, mannose; Glu, glucose; Gal, galactose; Ma-ol, mannitol; IS, internal standard.

Moreover, the monosaccharide components of the *Dendrobium* CPs were also analyzed by GC-MS (Figure 3). The results showed these polysaccharides mainly contain glucose and mannose, and minor amount of galactose.Tthe samples from Yunnan Province contained especially high ratios of glucose (Table 1).

### 2.2. Effects of Polysaccharides from Dendrobium on Macrophage Proliferation and Nitric Oxide Production

**Figure 4:**
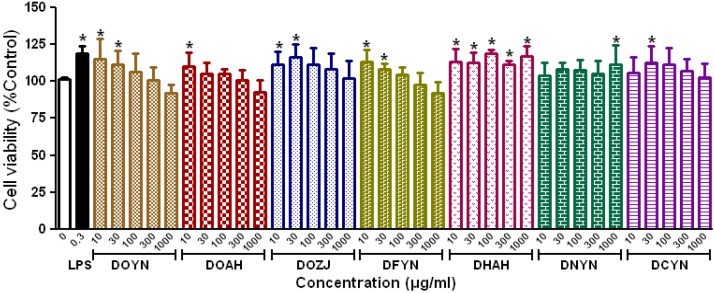
Effects of crude polysaccharides from *Dendrobium* on RAW 264.7 macrophagesproliferation.

^a^ Data, expressed as percentage of absorbance ratio between treatment and control, were shown as mean ± SEM of three independent experiments. * p < 0.05 *vs.* vehicle control.

Cell viability of mouse macrophages treated with a series of concentrations of CPs from *Dendrobium* and 0.3 μg/mL lipopolysaccharide (LPS) for 24 h were examined by the MTT method. The results showed that CPs from *Dendrobium* had no obvious cytotoxicity towards RAW 264.7 cells at the concentration of 1,000 μg/mL after 24 h culture. Actually, all CPs at low concentration, except CPs from DNYN, showed a certain promotion of macrophage proliferation (*p* < 0.05, Figure 4). 

The release of nitric oxide (NO) from RAW 264.7 cells stimulated by CPs from *Dendrobium* was detected. As shown in Table 2, CPs from DO exerted a significant stimulatory ability on NO production of macrophages (*p* < 0.05), specially CPs from DOYN which was the most potent. CPs from DF, DN and DC had lower ability to induce NO release from macrophages (*p* < 0.05).

**Table 2:** Effects of crude polysaccharides from *Dendrobium* on NO release from RAW 264.7 macrophages.

Samples	Concentrations (μg/mL)						
	0	0.3	10	30	100	300	1000
Control	0.8 ± 0.4						
LPS		100.2 ± 1 **					
DOYN			1.7 ± 0.7	6.3 ± 2	30.2 ± 7.7 *	68.8 ± 10.8 *	102.3 ± 8.9 **
DOAH			0.7 ± 1.3	2.5 ± 1.1	13.4 ± 3.1 *	34.9 ± 6.3 *	69.3 ± 9.4 **
DOZJ			0.5 ± 0.7	1 ± 1.1	3.3 ± 1.4	11.5 ± 2.9 *	36.9 ± 8.9 **
DFYN			ND	ND	ND	6.7 ± 1.8 *	31.2 ± 2.2 **
DHAH			ND	ND	1.1 ± 0.6	1 ± 1.2	2.2 ± 2
DNYN			ND	ND	ND	1.9 ± 1	13.5 ± 0.5 **
DCYN			ND	ND	1.1 ± 0.8	1.6 ± 1.9	6.8 ± 2.1 *

^a^ Data, shown as mean ± SEM, expressed as percentage of absorbance ratio between treatment and LPS, of at least three independent performed in quadruplicates for each sample. ^b^ ND: Not detectable. * *p* < 0.05, ** *p* < 0.001 *vs.* vehicle control.

### 2.3. Effects of Polysaccharides from Dendrobium on Phagocytosis

Flow cytometry was used to analyze the FITC-bead ingestion of macrophages in the different treated groups. The results indicated that LPS (0.3 μg/mL) and all CPs of *Dendrobium* at the tested concentrations could promote the phagocytosis activity of macrophages (*p* < 0.05) (Table 3). Especially, CPs from DOYN and DOAH showed similar effects on macrophage phagocytosis induced by 0.3 μg/mL LPS, which was a more than 2-fold increase compared to untreated cells (*p* < 0.05). 

**Table 3:** Effects of crude polysaccharides from *Dendrobium* on phagocytosis of RAW 264.7 macrophages.

Samples	Concentrations (μg/mL)				
	0	0.3	100	300	1000
Control	100				
LPS		269.7 ± 11 **			
DOYN			204.5 ± 35 *	233.7 ± 32.9 *	249.4 ± 30 *
DOAH			204.5 ± 21.5 *	207.9 ± 21.2 *	222.1 ± 26.4 *
DOZJ			196.3 ± 22.4 *	216.1 ± 19.7 *	213.9 ± 18.9 *
DFYN			165.6 ± 18.5 *	174.8 ± 20.9 *	192.9 ± 27.2 *
DHAH			170 ± 9.9 *	203 ± 6.7 **	208.6 ± 14.5 *
DNYN			181.6 ± 16.4 *	204.9 ± 12.2 *	214.6 ± 17.2 *
DCYN			159.9 ± 8 **	168.5 ± 18.9 *	210.1 ± 26.7 *

^a^ Data, expressed as percentage of absorbance ratio between treatment and control, were shown as mean ± SEM of three independent experiments. * p < 0.05, ** *p* < 0.001 *vs.* vehicle control.

### 2.4. Effects of Polysaccharides from Dendrobium on Cytokines Release of Macrophages

The releases of interleukin (IL)-1α, IL-6, IL-10 and tumor necrosis factor (TNF)-α from RAW 264.7 macrophages induced by CPs from *Dendrobium* are shown in Table 4. It was found that all CPs from *Dendrobium* could significantly induce TNF-α release of macrophages (*p* < 0.05), and CPs from DOYN was the most potent (*p* < 0.001) and even higher than that of LPS (*p* < 0.05).

**Table 4:** Effects of crude polysaccharides from *Dendrobium* on the secretion of cytokines.

Samples	Concentration (µg/mL)	Levels of cytokines (pg/mL)			
		IL-1α	IL-6	IL-10	TNF-α
Control	0	13.3 ± 1.7	6 ± 1	1.6 ± 0.5	4667 ± 1069.9
LPS	0.3	232 ± 31.5 **	12248.1 ± 168.6 **	1038.2 ± 41.5 **	19372 ± 1021.3 **
DOYN	100	55.5 ± 7.7 *	3484.7 ± 857.6 *	594.2 ± 18.3 **	9292.1 ± 189.2 *
	300	108.2 ± 16.8 **	5712.8 ± 920.7 **	858.7 ± 38.6 **	17711 ± 571.4 **
	1000	247.7 ± 19.7 **	11869.9 ± 2828.8 *	1328 ± 92.2 **	23323.3 ± 103 **
DOAH	100	32.1 ± 6.3 *	481.3 ± 27.6 **	168.2 ± 17 **	13694 ± 393.9 **
	300	132.8 ± 21.9 **	533.7 ± 99.1 **	457.3 ± 89.2 **	15940.8 ± 1081.5 **
	1000	167.9 ± 32.7 *	3291 ± 428.9 **	650.7 ± 77.9 **	16972.8 ± 871.5 **
DOZJ	100	ND	24.6 ± 7.8 *	98.6 ± 13.1 **	14104.1 ± 522.8 **
	300	19 ± 7.8	58.4 ± 10.6 *	117.9 ± 22.8 **	16377.8 ± 483.2 **
	1000	132.7 ± 32.3 *	1847.8 ± 91.6 **	588.5 ± 92 **	19078.2 ± 120.5 **
DFYN	100	3.2 ± 2.2	ND	ND	3671.6 ± 536.6
	300	4.6 ± 3.6	ND	8.8 ± 3.9	5900.6 ± 100.1
	1000	7.7 ± 1.9	52.1 ± 6.8 **	4.5 ± 0.4	9911.4 ± 356.1 *
DHAH	100	5.4 ± 1.9	ND	2.8 ± 0.8	12538.1 ± 831.4 **
	300	9.1 ± 1.3	13.1 ± 4.3	10.3 ± 3.9 *	14010.2 ± 54.4 **
	1000	18.6 ± 4.5	112.6 ± 16.1 **	16.1 ± 3.9 *	14963 ± 998.3 **
DNYN	100	9.5 ± 1.2	3.7 ± 0.7	ND	13902.8 ± 643.8 **
	300	32 ± 3 **	8 ± 5	7.5 ± 1.2	15741.3 ± 481.8 **
	1000	38.4 ± 5.1 *	17.1 ± 4.8 *	10.5 ± 1.1 **	16379.9 ± 465.3 **
DCYN	100	15.9 ± 1.2	ND	ND	4099.4 ± 284.5
	300	26.1 ± 2.7 *	ND	ND	7876.7 ± 88.1 *
	1000	41.7 ± 4.5 **	8 ± 5	ND	15354.3 ± 114.4 **

^a^ Data were shown as mean ± SEM of three independent experiments. ^b^ ND: Not detectable; * *p* < 0.05, ** *p* <0.001 *vs.* vehicle control.

CPs from DO, DH, DN and DC also induced IL-1α release from macrophages, but CPs from DOYN induced the highest level (*p* < 0.001). All CPs from DO (DOYN, DOAH, DOZJ), DF, DH and DN could certainly induce IL-6 release from macrophages (*p* < 0.05), but CPs from DC had no effect. However, only CPs from DO significantly induced higher IL-10 release from macrophages (*p* < 0.001). 

## 3. Discussion

Polysaccharides are present naturally in food ingredients and provide many benefits to the body. It has been well documented that polysaccharides from natural sources (mushrooms, algae, lichens and higher plants) are very potent macrophage immunomodulators [21,22]. In summary, polysaccharides can increase the cytotoxic activity of macrophages against tumor cells and microorganisms, activate phagocytic activity, increase reactive oxygen species and NO production, and enhance secretion of cytokines and chemokines, such as TNF-α, IL-1, IL-6, IL-8, IL-12, and IFN-γ. In this study, we found CPs from *Dendrobium* had promoting effects on macrophages, *i.e*., promoting macrophage proliferation, increasing phagocytic activity and inducing production of NO and TNF-α from macrophages. Among different species of *Dendrobium*, CPs from *D. officinale*, especially produced in Yunnan Province, exerted the strongest modulatory activities on the macrophage functions, suggesting that polysaccharides from *D. officinale* could be explored as novel potential immunomodulators for functional food development, which is in accordance with previous reports [15,16,23]. 

Up to now, the molecular mechanisms of the immunomodulatory activity of botanical polysaccharides on macrophage function have been deeply investigated. Specifically, botanical polysaccharides and/or glycoproteins usually bind to surface receptors (e.g., Toll-like receptor 4, CD14, complement receptor 3, scavenger receptor, dectin-1 and mannose receptor) in macrophages and induce similar immunomodulatory responses by subsequent activation of intracellular signaling cascades, resulting in transcriptional activation and production of immunomodulatory cytokines [21]. Several polysaccharides purified from *D. huoshanese* [5,6], *D. officinale* [23,24] and *D. denneanum* [8,25] have been proved to show immunomodulatory activity on macrophages, but their molecular mechanism remains unclear. It is reported that most of polysaccharides from *Dendrobium* were composed of β-(1→4) and (1→6)-linked mannose, β-(1→3)-linked mannose, β-(1→4)-linked mannose or glucose [5,26,27,28]. Our results also suggested that mannose was the major component of polysaccharides from *Dendrobium*. It was hypothesized that these *Dendrobium* polysaccharides might recognize the mannose/glucan receptor [29], dectin-1 and Toll-like receptor 2/4 and lead to activation of macrophage phagocytosis, oxidant production, endocytosis and nuclear factor-κB [21]. 

Generally the biological activities of polysaccharides are related to their chemical composition, configuration and chain conformation, as well as their physico-chemical properties. Polysaccharides from *Dendrobium* were different because of their different sugar components and ratios and/or chemical characteristics [18,19,20]. The molecular size of polysaccharides is an important physico-chemical parameter which may correlate with their biological activity. For example; the activity of (1→3)-β-glucans is strongly dependent on their Mw [30]. In the present study, though higher Mw CPs in *D.* *officinale* showed stronger stimulating activity on macrophages, the relationships between Mw and the activity in different species of *Dendrobium* were not clear, which may be attribute to their crude polysaccharides with various ratios of fractions with different Mw. Purified polysaccharides are necessary to clearly understand the effect of Mw on the bioactivity. Our previous chemical analysis on *Dendrobium* CPs’ carbohydrase enzymatic digestion properties and profiles revealed that the glycosidic linkages, such as 1,5-α-arabinofuranosidic, (1→4)-β-D-galactosidic, (1→4)-β-D-glucosidic, (1→4)-α-D-galactosiduronic and 1,4-β-D-mannosidic linkages were different in polysaccharides from *Dendrobium* [18,19]. The structure-activity features as well as high order structure of *Dendrobium* polysaccharides should be studied in future. 

## 4. Experimental

### 4.1. Materials

Seven samples of *Dendrobium*, including *D.* *officinale*, *D.* *fimbriatum*, *D.* *nobile* and *D.* *chrysotoxum* from Yunnan, *D.* *officinale* and *D.* *huoshanense* from Anhui and *D.* *officinale* from Zhejiang, were collected by us. The botanical origin of the material was identified by Professor Dong-xia Shen (China Pharmaceutical University and Yunnan Jinling Botanical Medicine Co., Ltd., Simao, China). The voucher specimens of *Dendrobium* were deposited at the Institute of Chinese Medical Sciences, University of Macau, Macao, China. Griess reagent, LPS and 3-(4,5-dimethylthiazol-2-yl)-2, 5-diphenyltetrazolium bromide (MTT) were obtained from Sigma-Aldrich (St. Louis, MO, USA). Dulbecco’s Modified Eagle Medium (DMEM), fetal bovine serum (FBS) and 1% penicillin/streptomycin (P/S) were purchased from Invitrogen Molecular Probes (Carlsbad, CA, USA). Rainbow fluorescent particles (3.0–3.4 μm) were purchased from BD Biosciences (San Diego, CA, USA), Milliplex Map kits were from Merck Millipore (Bedford, MA, USA). 

### 4.2. Preparation of Polysaccharides from Dendrobium

The reflux of powdered *Dendrobium* (5 g) was performed with distilled water (80 mL) for 60 min at 100 °C (FexIKA, Staufen, Germany). The extract solution was collected by centrifugation (4,000×g for 10 min, Allegra X-15R, Beckman Coulter, Indianapolis, IN, USA) and concentrated to 50 mL on a rotary evaporator (Büchi, Flawil, Switzerland). Then 50 mL extract solution was precipitated overnight (12 h) under 4 °C by addition of ethanol to a final concentration of 80% (v/v). The resulting precipitate was collected by centrifugation and washed twice with 95% ethanol (30 mL). After removal of ethanol on a water bath (60 °C), the residue was re-dissolved in hot (60 °C) water (200 mL) and then freeze dried (Coolsafe 110-4, Labogene ScanVac, Lynge, Denmark) to afford *Dendrobium* CPs. 1 mg/mL *Dendrobium* CPs was used for the phenol-sulfuric acid assay to determine the sugar content [31]. 

### 4.3. Polysaccharide Molecular Weight Determination

Average Mw and Mw distributions of the investigated polysaccharides were determined using an Agilent 1100 series LC system coupled with a DAWN EOS multi-angle laser light scattering photometer (MALLS, Wyatt Technology Co., Santa Barbara, CA, USA) and refractive index (RI) detector (G1362A, Agilent Technologies Inc., Santa Clara, CA, USA) [32]. In brief, 50 μL sample solution filtered through 0.22 μm nylon syringe filter was injected into the system, and separated at 40 °C on TSK G-6000PWXL (300 mm × 7.8 mm, i.d., 10 μm, Tosoh Bioscience, Tokyo, Japan) and TSK G-3000PWXL in series connected columns. Isocratic elution was performed with 15 mmol/L sodium chloride aqueous solution at a flow-rate of 0.5 mL/min. The [dn/dc] value for the tested samples was given as 0.140 mL/g. The data and chromatograms were recorded and processed by using ASTRA software (Wyatt Technology Co.). The DWAN EOS photometer was calibrated by using HPLC grade toluene (Merck, Whitehouse Station, NJ, USA) and normalized with a BSA standard (A9647, Sigma).

### 4.4. Compositional Monosaccharide Analysis of Polysaccharides

Compositional monosaccharide analysis of the investigated polysacharides was performed according to a previous report [33]. Briefly, dried samples after hydrolysis by TFA were treated with hydroxylamine hydrochloride-pyridine solution (1 mL, ~20 mg/mL) in a sealed glass tube equipped with a screw cap at 90 °C for 30 min, and then acetic anhydride (1 mL) was added and heating continued for another 30 min. The analysis was performed on an Agilent 6890 gas chromatography instrument coupled with an Agilent 5973 mass spectrometer (Agilent Technologies). A HP-5MS capillary column (30 m × 0.25 mm, i.d.) coated with 0.25 μm film 5% phenyl methyl siloxane was used for separation. The selected ion monitoring (SIM) method, *i.e.*, *m/z* 115 for ribose, arabinose, xylose, mannitol, *m/z* 129 for rhamnose and fucose, *m/z* 145 for mannose, glucose, galactose and *m/z* 168 for IS, was applied for accurate determination of the monosaccharides.

### 4.5. Cell Culture

RAW 264.7 mouse macrophage cells were obtained from American Type Culture Collection (Rockville, MD, USA) and cultured in DMEM medium supplemented with 10% FBS, 1% P/S at 37 °C in a humidified atmosphere of 5% CO_2_.

### 4.6. Cell Proliferation Assay

The viability of cells was measured using MTT assay. Briefly, 5 ° 10^3^ cells/well RAW 264.7 cells were plated in 96-well microplates overnight and then treated with serial concentrations of *Dendrobium* CPs or 0.3 μg/mL LPS for 24 h, respectively. Equal volume of medium was used as vehicle control. After treatment, cells were stained with MTT at final concentration of 0.5 mg/mL in PBS (pH 7.4) for another 4 h in dark and then the medium was discarded. The formazan crystals presented in cells were dissolved by 100 μL dimethyl sulfoxide. The absorbance was read at 570 nm on a microplate reader (1420 Multilabel counter victor^3^, Perkin-Elmer, Waltham, MA, USA). The results were expressed as ratio of absorbance values between treatment and vehicle control cells.

### 4.7. Phagocytic Activity Test

RAW 264.7 cells 20 × 10^4^/well were plated in 24-well plates overnight and then exposed serial concentrations of *Dendrobium* CPs for another 18 h. Equal volume of 0.3 μg/mL LPS or medium was used as the positive and vehicle control. After treatment, 5 μL rainbow FITC-fluorescent beads (about 1.0 × 10^7^ particles/well) were added and continually incubated for another 2 h in dark. Subsequently, cells were collected to a tube with 500 μL PBS and flow cytometry was used to count the phagocytized beads cells which displayed higher FITC fluorescence intensity. The phagocytosis activity was shown as percentage of macrophages ingested beads (BD FACSDiva Software V6.1.3). The results were expressed as ratio of phagocytic rate between treatment and vehicle control cells. 

### 4.8. Nitric Oxide Determination

RAW 264.7 cells 5 × 10^4^/well were seeded in 96-well microplates overnight,and then stimulated by serial concentrations of *Dendrobium* CPs or 0.3 μg/mL LPS for 24 h. Equal volume of medium was used to be vehicle control. After treatment, 75 μL of supernatants were collected and mixed with an equal volume of modified Griess reagent at room temperature for 15 min. The absorbance was measured at 540 nm with microplate reader. The NO production was expressed as ratio of absorbance values between treatment groups and LPS treated-group.

### 4.9. Quantitative Analysis of Cytokines

RAW 264.7 cells 5 × 10^4^/well were seeded in 96-well plates overnight and then exposed to serial concentrations of *Dendrobium* CPs or 0.3 μg/mL LPS for 24 h. The cell supernatants were collected by centrifugation at 1,000 × g for 10 min. The cytokines level (pg/mL) of IL-1α, IL-6, IL-10 and TNF-α in culture supernatant were measured by using a Luminex assay (Bio-Plex™ 200, Hercules, CA, USA) with commercially available Millilex Map kits according to the manufacturer’s instructions. In brief, 50 μL of standard of cytokines or test samples along with 50 μL mixed beads were added into the wells of a pre-wet 96-well filter plate and incubated overnight at 4 °C. After washing, 25 μL detection antibodies were added and incubated for 1 h at room temperature. Subsequently, 25 μL streptavidin-PE were added and incubated for another 30 min, and then washed. Finally, the beads were suspended in 150 μL assay buffer and analyzed by using Bio-Plex 200 instrument. The data were analyzed using Bio-Plex Manager™ software 5.0 (Bio-Rad).

### 4.10. Determination of Endotoxin Contamination

The endotoxin concentration in the *Dendrobium* CPs were tested by using Limulus Amebocyte Lysate assay (Lonza, Walkersville, MD, USA) with a Glucashield reconstitution buffer formulated to block interference of (1→3)-β-D-glucans (Associates of Cape Cod, Falmouth, MA, USA). In brief, the concentration of endotoxin in the test samples were calculated from the absorbance values of solutions containing known amounts of endotoxin standard. An *E. coli* O113:H10 endotoxin (Associates of Cape Cod) was used as endotoxin standard. The results indicated that the *Dendrobium* CPs contained less than 0.7 ng endotoxin/mg. That could exclude the possibility of endotoxin contamination in *Dendrobium* CPs.

### 4.11. Statistical Analysis

Results were expressed as mean ± SEM. Data were analyzed using one-way analysis of variance (ANOVA) followed by Turkey post-hoc test to determine the difference between groups (GraphPad Prism 5.0). Values of * *p* < 0.05 were considered as statistical significant. 

## 5. Conclusions

The effects of CPs from different species of *Detrobium* on macrophages functions were different. CPs in *D.* *officinale*, especially those collected from Yunnan Province, exerted the strongest immune-modulatory activities. Their diverse chemical characters might contribute to their varied effects on macrophage functions, which should be further investigated.

## References

[1] Yang L., Wang Z., Xu L. (2006). Simultaneous determination of phenols (bibenzyl, phenanthrene, and fluorenone) in *Dendrobium* species by high-performance liquid chromatography with diode array detection. J. Chromatogr. A..

[2] Ye Q., Qin G., Zhao W. (2002). Immunomodulatory sesquiterpene glycosides from *Dendrobium nobile*. Phytochemistry.

[3] Xu J., Zhao W.M., Qian Z.M., Guan J., Li S.P. (2010). Fast determination of five components of coumarin, alkaloids and bibenzyls in *Dendrobium* spp. using pressurized liquid extraction and ultra-performance liquid chromatography. J. Sep. Sci..

[4] Ng T.B., Liu J., Wong J.H., Ye X., Wing Sze S.C., Tong Y., Zhang K.Y. (2012). Review of research on *Dendrobium*, a prized folk medicine. Appl. Microbiol. Biotechnol..

[5] Zha X.Q., Luo J.P., Luo S.Z., Jiang S.T. (2007). Structure identification of a new immunostimulating polysaccharide from the stems of *Dendrobium huoshanense*. Carbohydr. Polym..

[6] Zha X.Q., Luo J.P., Jiang S.T. (2007). Induction of immunomodulating cytokines by polysaccharides from *Dendrobium huoshanense*. Pharm. Biol..

[7] Fan Y., He X., Zhou S., Luo A., He T., Chun Z. (2009). Composition analysis and antioxidant activity of polysaccharide from *Dendrobium denneanum*. Int. J. Biol. Macromol..

[8] Fan Y., Chun Z., Luo A., He T., He X. (2010). *In vivo* immunomodulatory activities of neutral polysaccharide (DDP1-1) from* Dendrobium denneanum*. Chin. J. Appl. Environ. Biol..

[9] Luo A., Song G., Chun Z., Qin J., Fan Y., He T. (2007). Inhibiting effect of tumor by *Dendrobium denneanum*. Chin. J. Appl. Environ. Biol..

[10] Luo A., Ge Z., Fan Y., Chun Z., Jin He X. (2011). *In vitro* and *in vivo* antioxidant activity of a water-soluble polysaccharide from *Dendrobium denneanum*. Molecules.

[11] Luo A., He X., Zhou S., Fan Y., He T., Chun Z. (2009). *In vitro* antioxidant activities of a water-soluble polysaccharide derived from *Dendrobium nobile* Lindl. extracts. Int. J. Biol. Macromol..

[12] Luo A., He X., Zhou S., Fan Y., Chun Z. (2010). Purification, composition analysis and antioxidant activity of the polysaccharides from *Dendrobium nobile* Lindl. Carbohydr. Polym..

[13] Luo A., Fan Y. (2011). Immune stimulating activity of water-soluble polysaccharide fractions from *Dendrobium nobile* lindl. Afr. J. Pharm. Pharmacol..

[14] Wang J.H., Luo J.P., Zha X.Q., Feng B.J. (2010). Comparison of antitumor activities of different polysaccharide fractions from the stems of *Dendrobium nobile* Lindl. Carbohydr. Polym..

[15] Wang J.H., Luo J.P., Zha X.Q. (2010). Structural features of a pectic polysaccharide from the stems of *Dendrobium nobile* Lindl. Carbohydr. Polym..

[16] Wang J.H., Luo J.P., Yang X.F., Zha X.Q. (2010). Structural analysis of a rhamnoarabinogalactan from the stems of *Dendrobium nobile* Lindl. Food Chem..

[17] Jin L.H., Liu C.F., Tang T., Shen L. (2010). Experimental study on anti-tumor effect of Dendrobium *candidum* polysaccharides. Chin. Pharm. J..

[18] Huang M.Q., Ruan J.Y. (1997). Monosaccharide composition analysis of 6 water-soluble polysaccharides from *Dendrobium* species. Zhongguo Zhong Yao Za Zhi.

[19] Xu J., Guan J., Chen X.J., Zhao J., Li S.P. (2011). Comparison of polysaccharides from different *Dendrobium* using saccharide mapping. J. Pharm. Biomed. Anal..

[20] Zha X.Q., Pan L.H., Luo J.P., Wang J.H., Wei P., Bansal V. (2012). Enzymatic fingerprints of polysaccharides of *Dendrobium officinale* and their application in identification of *Dendrobium* species. J. Nat. Med..

[21] Schepetkin I.A., Quinn M.T. (2006). Botanical polysaccharides: Macrophage immunomodulation and therapeutic potential. Int. Immunopharmacol..

[22] Tzianabos A.O. (2000). Polysaccharide immunomodulators as therapeutic agents: Structural aspects and biologic function. Clin. Microbiol. Rev..

[23] Xia L., Liu X., Guo H., Zhang H., Zhu J., Ren F. (2012). Partial characterization and immunomodulatory activity of polysaccharides from the stem of *Dendrobium officinale (Tiepishihu) in vitro*. J. Funct. Foods.

[24] Liu X.F., Zhu J., Ge S.Y., Xia L.J., Yang H.Y., Qian Y.T., Ren F.Z. (2011). Orally administered *Dendrobium officinale* and its polysaccharides enhance immune functions in BALB/c mice. Nat. Prod. Commun..

[25] Fan Y., Luo A. (2011). Evaluation of anti-tumor activity of water-soluble polysaccharides from *Dendrobium denneanum*. Afr. J. Pharm. Pharmacol..

[26] Hsieh Y.S.Y., Chien C., Liao S.K.S., Liao S.F., Hung W.T., Yang W.B., Lin C.C., Cheng T.J.R., Chang C.C., Fang J. M. (2008). Structure and bioactivity of the polysaccharides in medicinal plant *Dendrobium huoshanense*. Bioorg. Med. Chem..

[27] Hua Y.F., Zhang M., Fu C.X., Chen Z.H., Chan G.Y.S. (2004). Structural characterization of a 2-O-acetylglucomannan from *Dendrobium officinale* stem. Carbohydr. Res..

[28] Xu C., Chen Y.L., Zhang M. (2004). Structural characterization of the polysaccharide DMP2a-1 from *Dendrobium moniliforme*. Chin. Pharm. J..

[29] Ezekowitz R.A.B., Sastry K., Bailly P., Warner A. (1990). Molecular characterization of the human macrophage mannose receptor: Demonstration of multiple carbohydrate recognition-like domains and phagocytosis of yeasts in Cos-1 cells. J. Exp. Med..

[30] Zhang M., Cui S.W., Cheung P.C.K., Wang Q. (2007). Antitumor polysaccharides from mushrooms: a review on their isolation process, structural characteristics and antitumor activity. Trends Food Sci. Technol..

[31] Dubois M., Gilles K.A., Hamilton J.K., Rebers P.A., Smith F. (1956). Colorimetric method for determination of sugars and related substances. Anal. Chem..

[32] Xie J., Zhao J., Hu D.J., Duan J.A., Tang Y.P., Li S.P. (2012). Comparison of polysaccharides from two species of *Ganoderma*. Molecules.

[33] Guan J., Yang F.Q., Li S.P. (2010). Evaluation of carbohydrates in natural and cultured *Cordyceps* by pressurized liquid extraction and gas chromatography coupled with mass spectrometry. Molecules.

